# Overlapping rheumatoid arthritis and antisynthetase syndrome with secondary Sjögren’s syndrome: a case report and review of the literature

**DOI:** 10.1186/s13256-022-03353-3

**Published:** 2022-04-04

**Authors:** W. S. Weerasinghe, Chandrika Jayasinghe

**Affiliations:** 1 Teaching Hospital, Peradeniya, Sri Lanka; 2 Department of Medicine, Faculty of Medicine, Peradeniya, Sri Lanka

**Keywords:** Overlap syndromes, Rheumatoid arthritis, Antisynthetase syndrome, Sjögren’s syndrome

## Abstract

**Background:**

Overlap syndromes account for about 25% of autoimmune diseases. They are many possible combinations of known autoimmune diseases increasingly diagnosed with the identification of of a large number of autoantibodies. In this case report, we present a patient with rare overlapping rheumatoid arthritis–antisynthetase syndrome with associated secondary Sjögren’s syndrome atypically presenting without interstitial lung disease.

**Case presentation:**

A 52-year-old Sinhalese female, a known patient with type 2 diabetes mellitus, presented with a history of symmetrical inflammatory-type polyarthritis with significant morning stiffness, proximal muscle weakness, pain, and roughening of the fingertips with associated sicca symptoms of 5 months duration. Examination revealed features of active joint inflammation, mechanic’s hand, xerostomia, and left-sided breast lump. Investigations confirmed the presence of rheumatoid arthritis with strongly positive rheumatoid factor (202 U/ml) and anti-cyclic citrullinated peptide antibody (717 U/ml). Antisynthetase syndrome was also diagnosed with borderline-positive anti-aminoacyl-tRNA antibodies but without interstitial lung disease. Sjögren’s syndrome was confirmed by the clinical history and histology and considered a secondary disorder. As her breast lump proved to be benign, no further interventions were done. She was started on sulfasalazine and methotrexate with steroid bridging therapy and achieved remission and had good control of the disease without any joint deformity or flare-up on 6-month clinic follow-up.

**Discussion:**

Overlapping rheumatoid arthritis–antisynthetase syndrome is a very rare disease with disabling complications. Early identification of the atypical presentations of the overlap syndromes, by thorough investigations, helps physicians to prescribe proper disease-modifying antirheumatoid drugs and biological drugs. It also helps predict the prognosis of the patients before they develop complications.

## Background

Overlap syndromes account for about 25% of autoimmune diseases [1] and are increasingly diagnosed with the identification of a large number of autoantibodies. Early identification of overlap syndromes will help predict the outcome and decide the best treatment modality for these patients. This case report presents a patient with overlapping rheumatoid arthritis (RA)–antisynthetase syndrome (ASA) with secondary Sjögren’s syndrome (SS) atypically presenting without interstitial lung disease (ILD).

## Case presentation

A 54-year-old Sinhalese female who is a known patient with type 2 diabetes mellitus for 2 years presented with a history of left-sided (L/S) ankle joint (AJ) pain and swelling starting 5 months ago. A month later, she developed right-sided (R/S) AJ pain followed by L/S knee joint pain and multiple small joints of both hands with some morning stiffness lasting approximately 1–2 hours each day. While joint pain was progressing, she also developed dryness of the mouth and eyes, which became prominent 2 months before presentation. For 2 months she also had pain in the bilateral calf and thigh with difficulty getting up from the seated position. For 1 month, she noticed some roughening of the tips and sides of her fingers. She also had constitutional symptoms including generalized malaise, loss of weight, and loss of appetite. At the time of presentation, she had R/S knee joint (KJ) pain, pain in the small joints of the hands, L/S calf pain, dry eyes, and dry mouth with difficulty swallowing solid foods. She did not have excessive loss of hair oral ulcers or photosensitive rashes. There was no skin tightening in the hands, finger discoloration, recurrent oral ulcers, hair loss, photosensitivity, or any other skin rashes. Moreover, there was no significant weight gain, cold intolerance, or any other features of hypothyroidism, and bowel habits were normal.

She did not have a history suggestive of venous thrombosis or pregnancy loss. Her diabetes was well controlled with oral hypoglycemic, and no evidence of macro- or microvascular complications was evident in history. There was no family history of autoimmune disease. She had undergone two lower (uterine) segment cesarean sections (LSCS), appendectomy, paraumbilical hernia repair (PUH), and total abdominal hysterectomy (TAH) previously without any anesthetic complications.

**Fig. 1:**
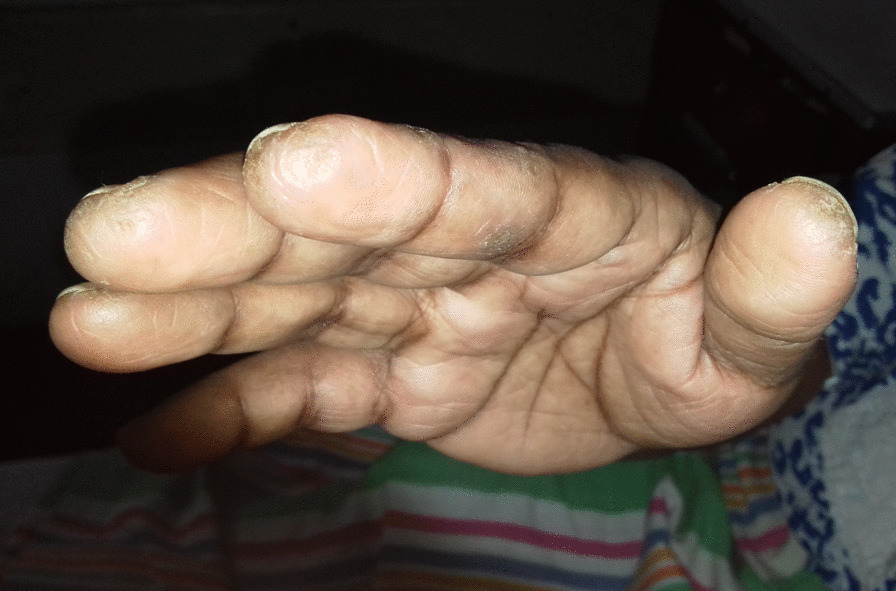
Mechanic’s hands with fissures and roughness with hyperkeratosis and scaling on the pulp of the thumb and the radial aspect of the index finger

On examination, she was of average build and her lips were dry with white depositions in gums but no parotid swelling. Thickened skin over the tips and sides of her fingers was compatible with the mechanic’s hand sign (Fig. 1). No other skin manifestations of autoimmune disease were found, including Gottron papules or malar rash. She was not pale or icteric and had no hepatosplenomegaly, lymphadenopathy, or parotid swelling.

Her musculoskeletal examination revealed mild tenderness on her L/S KJ, metacarpophalangeal joints (MCP), and proximal interphalangeal joints (PIP) of both hands, which sum up to a Disease Activity Score 28 (DAS 28) of 6.90, indicating severe disease activity. Muscle examination using Manual Muscle Testing Grading system (MMT) showed an asymmetric proximal myopathic pattern in bilateral lower limbs (left hip flexion 3+, right hip flexion 4, and hip extension 5 bilaterally) without any small muscle involvement in both upper and lower limbs.

Her breast examination revealed a firm-to-soft small mobile lump at the left breast with no skin changes or axillary lymphadenopathy suggestive of malignancy. She had a heart rate 88 beats per minute, blood pressure 110/70 mmHg, and normal first and second heart sounds. Her systemic examination was normal without any fine bibasal crepitation, organomegaly, or neuropathy.

Her Initial investigations are summarized below (Table 1). Her blood panel revealed high total white blood cell count (WBC) with mild eosinophilia and few atypical lymphocytes, normochromic normocytic red cells, and rouleaux formation. Her erythrocyte sedimentation rate (ESR) 125 mm per hour, rheumatoid factor (RhF) 202 U/ml, and anti-cyclic citrullinated peptide antibody level (anti-CCP) 717 U/ml confirmed rheumatoid arthritis. As part of the diagnostic workup, screening for hepatitis B, hepatitis C, and human immunodeficiency virus (HIV) serologies  were done and found to be negative.

**Table 1:** Initial investigations

Investigation	Results
White blood cell count (WBC)	11.4 × 10^9^/L (neutrophils 51%, lymphocytes 31%)
Hemoglobin (Hb)	13.1 g/dL
Mean corpuscular volume (MCV)	83.9 fL
Platelets (PLT)	397 × 10^9^/L
Aspartate aminotransferase (AST)	31.5 U/L
Alanine aminotransferase (ALT)	30.8 U/L
Serum creatine (SCr)	75 μmol/L
Blood urea (BU)	3.2 mmol/L
Serum albumin (Alb)	40.1 g/dL
Serum sodium (Na)	140 mmol/L
Serum potassium (K)	4.46 mmol/L
Creatine phosphokinase (CPK)	47 μg/L (normal range 24–190 μg/L)
Creatine kinase myocardial band (CK-MB)	19 IU/L (normal range 1.8–24 IU/L)
Erythrocyte sedimentation rate (ESR)	125 mm per hour
C-reactive protein (CRP)	42 mg/L
Rheumatoid factor (RhF)	202 U/ml
Anti-cyclic citrullinated peptide antibody level (anti-CCP)	717 U/ml (< 25 U/ml)
Thyroid-stimulating hormone (TSH)	3.611 μIU/ml

Electrocardiogram (ECG), chest x-ray, urine full report, and clotting profile were normal

**Fig. 2:**
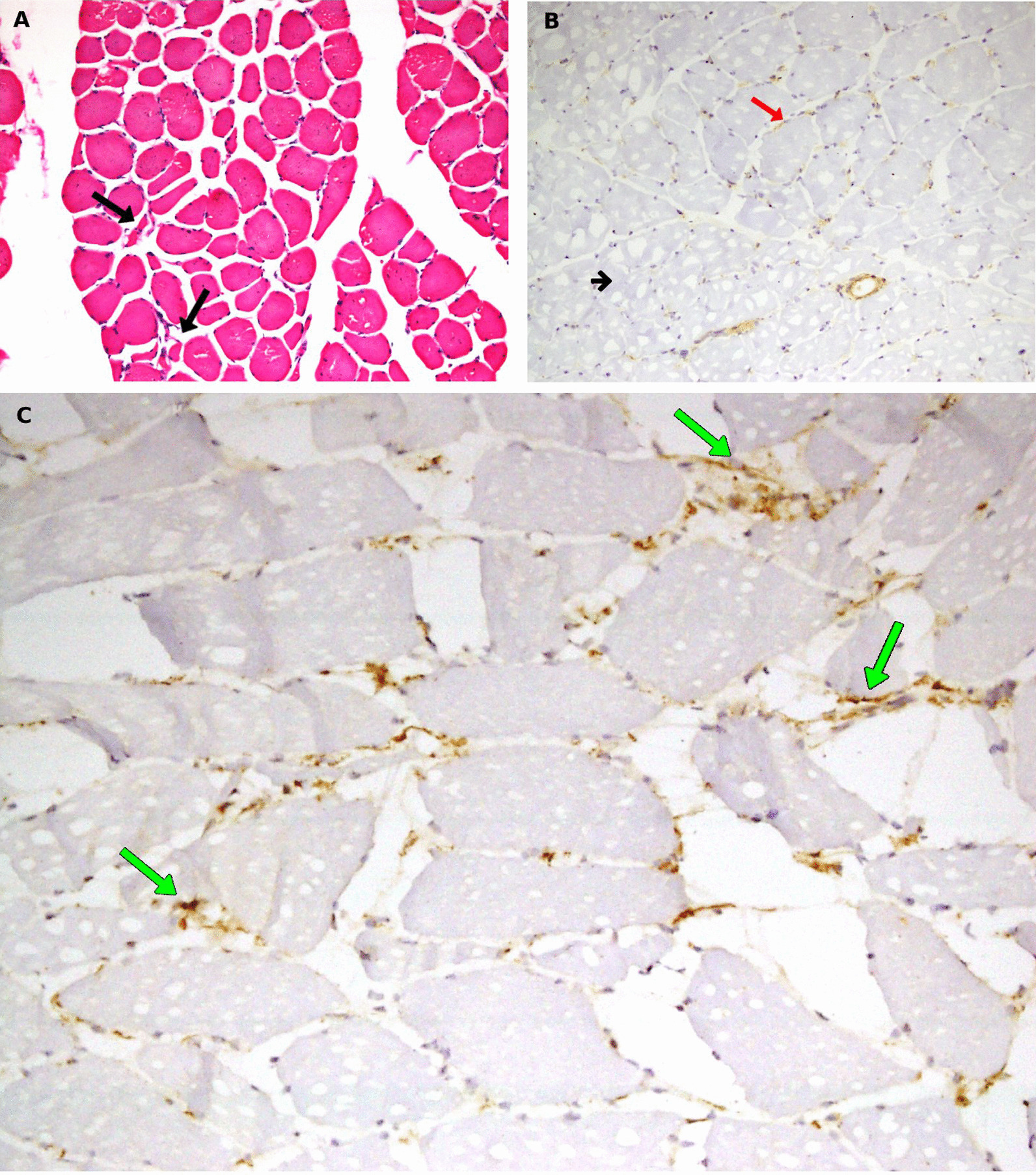
Muscle biopsy. Hematoxylin and eosin stain (×20) showing occasional atrophic muscle fibers seen between normal muscle fibers (atrophic fibers indicated by black arrows) (**A**). Muscle biopsy immunoperoxidase stain (×10) showing depleted major histocompatibility complex (MHC) class 1 expressing capillaries in muscle tissues (short black arrow) and normal muscle tissues with MHC class 1 expression (red arrow) (**B**). Muscle biopsy immunoperoxidase stain (×10) showing deposition of C5b9 complement complexes in capillaries in the muscle tissues (green arrows) (** C**)

Electromyography (EMG) showed fibrillatory potentials of short duration and small amplitude, as well as polyphasic motor unit action potentials (MUAPs) with early recruitment consistent with myositis. Muscle biopsy showed occasional atrophic fibers, focal reduction of major histocompatibility complex (MHC) class 1 stain, and deposits of C5b9 membrane complex in the capillaries; observations are consistent with early changes of dermatomyositis (Fig. 2A–C).

An extractable nuclear antigen panel (ENA) was done (immunoblot method), revealing borderline-positive anti-aminoacyl-tRNA antibodies (anti-Jo-1), and anti-double-stranded DNA (DS DNA), anti-Sjögren syndrome A-native (60 kDa) (SSA), RO-52 recombinant, anti-Sjögren syndrome B (SSB), antibodies directed against the U1 ribonucleoprotein/anti-Smiths (RNP/SM), anti-PM/Scl antibodies, centromere B (CB), proliferating cell nuclear antigen (PCNA), nucleosome (NUC), histone (HI), ribosomal-P-protein antibody, and anti-mitochondrial M2 antibody (AMA-M2) were negative.

Her lung functions were assessed for possible ILD. Chest x-ray was normal, oxygen saturation (SpO_2_) was 96% at rest and 98% after 6 minutes walking (408 m), spirometry was normal [FEV1/FVC 83, FEV1 1.86 L (108%), FVC—2.24 L (110%), FEV25-75 68%]. As the lung function test was normal and the patient was not symptomatic, high-resolution computed tomography (HRCT) was not done. Considering the symptoms and the autoantibody profile, a diagnosis of ASA overlap with RA was made.

**Fig. 3:**
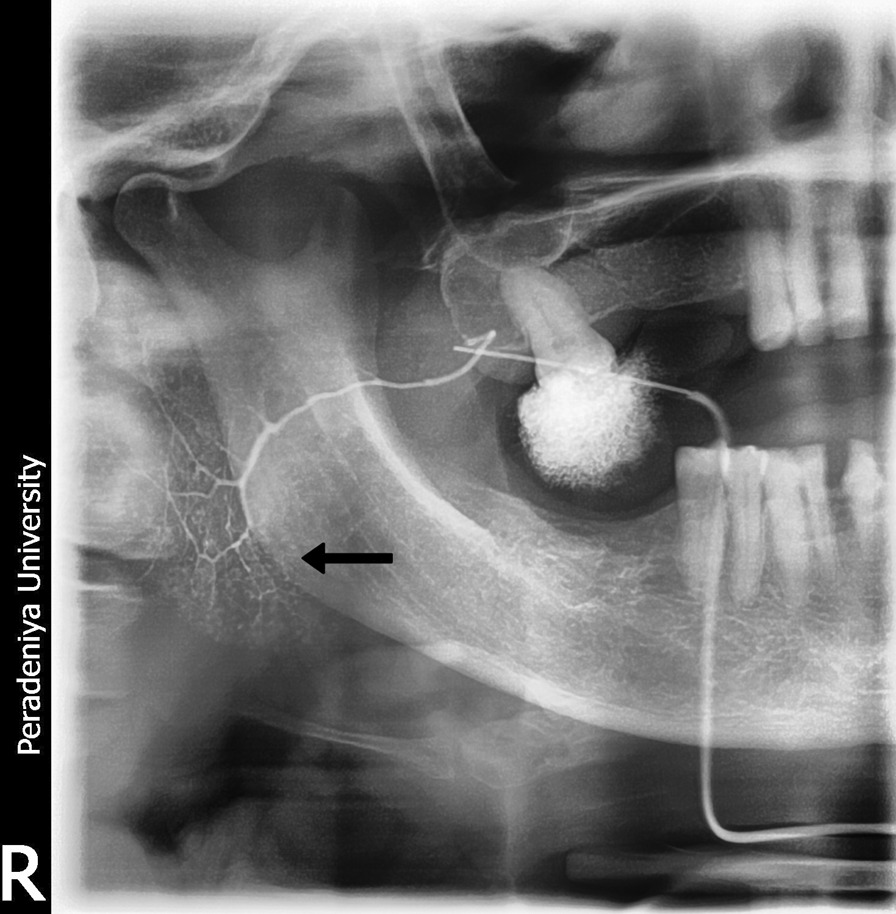
Sialogram showing punctate sialectasis (black arrow), dots, and blobs of contrast media within the salivary gland or “snowstorm appearance”

**Fig. 4:**
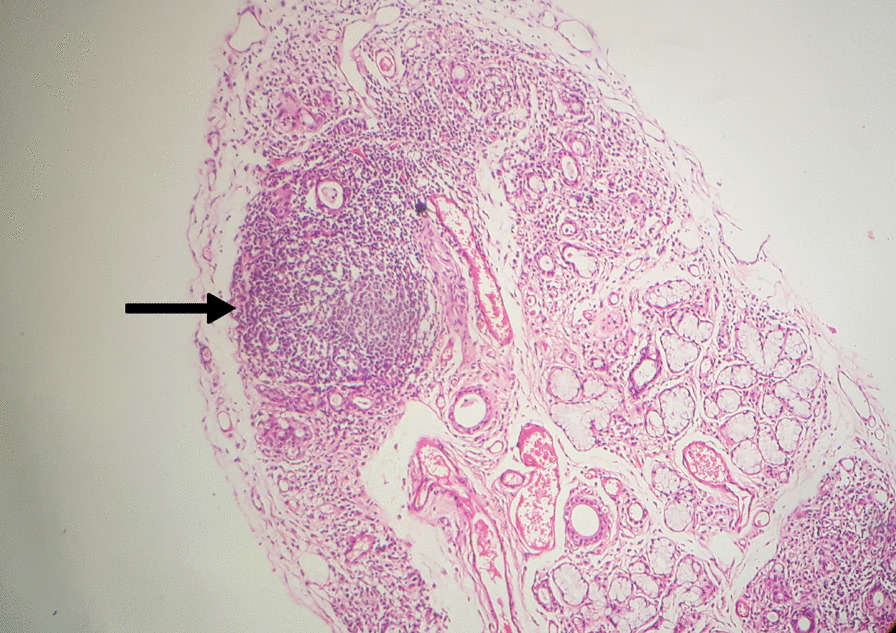
Salivary gland biopsy showing minor salivary tissue containing a dense localized periductal lymphoplasmacytic infiltrate in a few foci with epimyoepithelial islands (black arrow)

She was also evaluated for SS and was found to have a positive Schirmer's test and reduced saliva flow on sialometry and sialogram (Fig. 3). A minor salivary gland biopsy of the lower lip showed minor salivary tissue containing a dense localized periductal lymphoplasmacytic infiltrate in a few foci with epimyoepithelial islands confirming SS; however, the Focus Score (FS), which correlates with the disease activity, was not given in the histology report (Fig. 4).

Serum protein electrophoresis showed a mild polyclonal increase in gamma globulins and no abnormal monoclonal bands. Her chest x-ray was normal, and her x-ray hands showed no calcifications of deformities. Ultrasound abdomen was normal with no organomegaly or lymphadenopathy. Two-dimensional echocardiogram was normal with ejection fraction (EF) of > 60%.

Her breast lump was evaluated with mammography, showing a mass at left breast 11 o’clock position that could represent a fibroadenoma (typical), a focal asymmetry at the upper outer quadrant of the right breast (RUOQ) with a corresponding complex cystic lesion on ultrasound scan (USS) [Breast Imaging Reporting and Data System (BIRADS) IVa: low level of suspicion for malignancy], and fine pleomorphic group calcification in lower inner quadrant of the right breast, suggesting true cut biopsy and magnetic resonance imaging (MRI) for further evaluation. MRI of the breast showed two solid lesions, likely benign, at the 10 o’clock position of the right breast and 9 o’clock position of the left breast. The true cut biopsy was unsuccessful, but further assessment was not done owing to the benign nature of the lump after a discussion with radiologists and the surgical team.

After confirming the diagnosis of overlapping rheumatoid arthritis–antisynthetase syndrome and secondary Sjögren’s syndrome, she was started on methotrexate (MTX) 7.5 mg weekly and sulfasalazine 500 mg three times daily (tds) with oral prednisolone 40 mg/day bridging therapy and tailed off later. A course of oral amoxicillin 500 mg tds, metronidazole 400 mg tds for 3 days was given, and long-term 0.2% chlorhexidine mouthwash 10 ml two times daily (bd) and pilocarpine eye drops were also prescribed for the SS by the oral and maxillofacial surgery team. Her MTX dose increased according to the response and disease activity monitored at the medical clinic, and her DAS 28 score decreased from 6.90 to 2.40 within 4 months of treatment, indicating remission. Her muscle power improved to MMT grade 5, but we were unable to repeat the shimmers test or salivary gland biopsy, yet she showed remarkable improvement of her xerostomia, and parotid swelling. Owing to her good compliance, the disease was in remission without the development of any joint deformity on 6-month follow-up visit.

## Discussion

Inappropriate or maladaptive host immune response to either external or internal triggers can generate a wide variety of diseases that we call autoimmune diseases [2]. Five major diffuse connective tissue entities have been identified: systemic lupus erythematosus (SLE), systemic sclerosis (SSC), polymyositis (PM), dermatomyositis (DM), and rheumatoid arthritis (RA). A sixth disorder, Sjögren’s syndrome (SS), is commonly associated with each of these diseases but is called primary SS when it occurs alone. If at least two connective tissue diseases are identified by satisfying classification criteria and occurring at the same time in one patient, the definition of overlap syndrome (OS) is fulfilled [3]. OS accounts for 25% of all connective tissue disorders [1], and there are two approaches of classification that are practiced in clinical medicine. Depending on the clinical features of the diseases overlapped and the specific antibody marker, they are subdivided into either a specific entity like ASA or can be identified as a pattern of clinical features without a specific serologic marker [4]. These classifications are very arbitrary, and occurrence of overlap among these two groups was also found [5]. However, the treatment depends on the most prominent disorder.

Our patient was diagnosed with RA owing to the fulfillment of the 2010 American College of Rheumatology/European League Against Rheumatism Collaborative Initiative criteria (ACR/EULAR) [6]. She was also diagnosed with ASA owing to her arthritis, polymyositis, mechanic’s hands (Fig. 1), positive anti-JO-1 antibody, and fulfillment of the criteria proposed by Connors *et al*. [7] and Solomon *et al*. [8]. Sjögren’s syndrome was also diagnosed by clinical features, positive shimmers test, sialometry, sialogram (Fig. 4), exclusion of other mimicking diseases, and histology of minor salivary gland biopsy (Fig. 3), fulfilling the revised version of the European criteria proposed by the American-European Consensus Group [9]. These findings are compatible with an overlap syndrome comprising rheumatoid arthritis, ASA, and probable secondary Sjögren’s syndrome. The most unique feature of our case is this rare combination of disease and the atypical presentation of the ASA.

Although ASA, by definition, has an element of arthritis, it is important to clarify the possibility of an overlap disease because our patient did not have any respiratory symptoms or radiological evidence of ILD, which is the main component of ASA. Some treatment decisions like starting anti-tumor necrosis factor (anti-TNF) drugs may be affected by subcategorization of the disease components. RA is a common chronic autoimmune disease with symmetrical, peripheral polyarthritis, evolving over a few weeks or months and characterized by widespread persistent synovitis (inflammation of the synovial lining of joints, tendon sheaths, or bursae) and positivity autoantibodies to the Fc portion of immunoglobulin G (rheumatoid factor) and anti-CCP. Overexpression of tumor necrosis factors (TNF) was recognized as a key inflammatory element in RA causing joint inflammation and deformity [10]. Presentation of the diseases is also very heterogeneous in some patients and has been found to be associated with other autoimmune diseases, including Sjögren’s syndrome. This patient’s arthritis may be categorized as a part of ASA, but the presence of the anti-CCP antibody in a very high titer is a very specific and prognostic indicator for RA (> 95%). Meyer *et al*. found that these patients can develop more severe and erosive arthritis after a 7-year median follow-up compared with patients with ASA alone. Interestingly, they also observed the possibility of ASA flair-up when treated with anti-TNF drugs [5]. This shows the importance of identifying the component of OS to adapt the best treatment modality. This case also emphasizes the importance of measuring anti-CCP antibody levels in overlap diseases associated with RA, which impact the treatment decisions. RA was considered the predominant entity of this patient because she did not have classic ILD associated with ASS and she had a high anti-CCP titer, which has very high specificity for RA [11]. A case–control retrospective study of patients with anti-CCP (*n* = 7) first identified in a French nine-center registry of 284 patients with ASS, found the presence of anti-CCP in ASA, which the authors categorized as overlapping RA–ASA syndrome [5]. In the absence of ILD, the treatment was mainly guided by the RA component and was done according to the ACR/EULAR guidelines.

Our patient had an atypical presentation of ASA. Though ASA is a variant of polymyositis associated with polymyositis that was first reported in 1976 [12], it belongs to a family of rare systemic autoimmune diseases and there is a classical triad of myositis, ILD, and arthritis associated with the presence of the antibody directed against tRNA synthetase (an enzyme involved in protein synthesis in many tissues, including muscle and lung) [13]. In addition to the many anti-JO-1 antibodies, which occur in 80% of patients with ASA [14], a wide variety of other autoantibodies (20%) are associated with antisynthetase syndrome and are presented in Table 2 in the order of prevalence [3, 7, 14]. Our patient also had muscle symptoms, and EMG and muscle biopsy showed classic features of myositis, which is more in favor of dermatomyositis, confirming the myositis despite CPK level being normal, which is another interesting feature of this case [13]. Schmidt *et al*. showed that myositis was found in 91% of subjects, alveolitis in 75%, arthritis in 74%, Raynaud’s phenomenon in 50%, carpal tunnel syndrome in 37%, sicca symptoms in 34%, and tenosynovitis in 31% in a cohort of 200 anti-Jo-1-positive patients [15]. Out of the three minor criteria for diagnosing ASA, arthritis and mechanic’s hands were present in our patient, confirming the diagnosis of ASA [8].

**Table 2:** Antisynthetase antibodies

Antibody	Antigen (tRNA synthetase)
JO-1	Histidyl
PL-7	Threonyl
PL-12	Alanyl
EJ	Glycyl
OJ	Isoleucyl
KS	Asparaginyl
Ha	Tyrosyl
Wa	48-kDa transfer RNA-related protein
Zo	Phenylalanyl

RNA = ribonucleic acid; tRNA = transfer ribonucleic acid; kDa = kilodalton

ILD is one of the most common presentations of ASA (90% associated) [13], the commonest being nonspecific interstitial pneumonia (NSIP) pattern [16]. Hence, the lack of clinical features of ILD is an atypical presentation. Some studies also found that ILD preceded myositis in clinical presentation in a minority of cases [16, 17]. There were also reports of incorrect diagnosis as idiopathic pulmonary fibrosis (IPF) due to subtle manifestations of ASA leading to incorrect treatment approaches [16]. Our patient was screened at the chest clinic for ILD by a chest physician by a thorough clinical examination and underwent lung function tests using spirometry, and both were normal. Owing to negative screening tests, we did not perform urgent high-resolution CT chest (HRCT) considering the radiation exposure, cost, and the local protocols of the hospital. Connors *et al*. proposed an algorithm for investigation that also did not emphasize doing HRCT in a patient with a normal spirometry test [7]. However, very early lung involvement cannot be excluded by the screening tests, so she required follow-up [16, 18]. Moreover, the detection of very early lung involvement by invasive tests will not alter the treatment as corticosteroids with or without disease-modifying antirheumatoid drugs (DMARDs) are the first treatment option [16, 18, 19]. Since ILD is an important prognostic marker of the disease, regular follow-up at the chest clinic was arranged, aiming for early identification [20].

We started her on MTX as it was widely available and recommended first-line treatment of RA, then escalated the dose accordingly while monitoring disease activity using DAS 28 score. Theoretically, she is at high risk of developing erosive arthritis if the disease  dose not comes into remission with DMARDs. Hence, early introduction of biological drugs like anti-TNF, anti-CD20 monoclonal antibodies should be considered in patients at first relapse according to current guidelines. However, anti-TNF drugs may be avoided in this case owing to possible ASA flair-up. Luckily, our patient did well with DMARDs, so biologics were not started. The other very important step in management is the close and regular monitoring of the disease activity and complications. Though her lungs were fine on initial evaluation, she is at a very high risk of developing ILD in the future, which warrants meticulous follow-up under a chest physician for early identification and proper management of disabling ILD for which we have referred her. SS was well controlled with the initial supportive measures, including artificial tears and DMARDs, so she did not suffer adverse consequences.

## Conclusion

We conclude that early identification of the disease components of the overlap syndromes, by thorough investigations, helps physicians to guide DMARDs and biological drugs according to the most active disease. Unique and rare combinations like this can cause diagnostic problems, especially when they present in an atypical manner. It is very important to titrate treatment guided by the current evidence and expected complications such as ILD because some treatments can worsen some disease components while curing others. Complete and precise diagnosis also helps predict the prognosis of the patients before they develop disabling complications. Autoimmune diseases are complex; though guidelines propose specific criteria to diagnose them, we commonly encounter nonspecific symptoms and signs. We hypothesize that overlap diseases are more common than the available figures in literature, and to diagnose them clinicians need to perform careful clinical examination and targeted in-depth investigations.

## Data Availability

Not applicable.

## Abbreviations

ASA: Antisynthetase syndrome
RA: Rheumatoid arthritis
SS: Sjögren’s syndrome
ILD: Interstitial lung disease
L/S: Left-sided
AJ: Ankle joint
R/S: Right-sided
KJ: Knee joint
LSCS: Lower (uterine) segment cesarean sections
PUH: Paraumbilical hernia repair
TAH: Total abdominal hysterectomy
MCP: Metacarpophalangeal joints
MMT: Manual Muscle Testing Grading system
PIP: Proximal interphalangeal joints
WBC: White blood cell count
RhF: Rheumatoid factor
Anti-CCP: Anti-cyclic citrullinated peptide antibody level
MHC: Major histocompatibility complex
EMG: Electromyography
MUAPs: Motor unit action potentials
Hb: Hemoglobin
MCV: Mean corpuscular volume
PLT: Platelets
AST: Aspartate aminotransferase
ALT: Alanine aminotransferase
SCr: Serum creatine
BU: Blood urea
Alb: Serum albumin
Na: Serum sodium
K: Serum potassium
CPK: Creatine phosphokinase
CK-MB: Creatine kinase myocardial band
ESR: Erythrocyte sedimentation rate
CRP: C-reactive protein
TSH: Thyroid-stimulating hormone
ENA: Extractable nuclear antigen panel
Anti-JO-1: Anti-aminoacyl-tRNA antibodies
DS DNA: Anti-double-stranded DNA
SSA: Anti-Sjögren syndrome A-native (60 kDa)
SSB: Anti-Sjögren syndrome B
RNP/SM: Antibodies directed against the U1 ribonucleoprotein/anti-Smiths
CB: Centromere B
PCNA: Proliferating cell nuclear antigen
NUC: Nucleosome
HI: Histone
AMAM2: Anti-mitochondrial M2 antibody
FEV1: Forced expiratory volume
FVC: Forced vital capacity
FEV25-75%: Forced expiratory flow at 25% and 75% of the pulmonary volume
HRCT: High-resolution computed tomography
RUOQ: Upper outer quadrant of the right breast
USS: Ultrasound scan
BIRADS: Breast Imaging Reporting and Data System
MRI: Magnetic resonance imaging
MTX: Methotrexate
DAS 28: Disease Activity Score 28
SLE: Systemic lupus erythematosus
SSC: Systemic sclerosis
PM: Polymyositis
DM: Dermatomyositis
OS: Overlap syndrome
ACR/EULAR: American College of Rheumatology/European League Against Rheumatism
Anti-TNF: Anti-tumor necrosis factor
NSIP: Nonspecific interstitial pneumonia
